# Bellidifolin Inhibits SRY-Related High Mobility Group-Box Gene 9 to Block TGF-*β* Signalling Activation to Ameliorate Myocardial Fibrosis

**DOI:** 10.1155/2022/6841276

**Published:** 2022-05-09

**Authors:** Ting-Ting Yao, Hong-Xia Yang, Jia-Huan Sun, Yue Zhang, Yu Zhang, Qiu-Hang Song, Wei-Zhe Liu, Juan-Juan Zhang, Ai-Ying Li

**Affiliations:** ^1^Department of Biochemistry and Molecular Biology, College of Basic Medicine, Hebei University of Chinese Medicine, Shijiazhuang 050200, Hebei, China; ^2^Hebei Key Laboratory of Chinese Medicine Research on Cardio-cerebrovascular Disease, Shijiazhuang 050091, Hebei, China; ^3^Hebei Higher Education Institute Applied Technology Research Center on TCM Formula Preparation, Shijiazhuang 050091, Hebei, China; ^4^College of Nursing, Hebei University of Chinese Medicine, Shijiazhuang 050091, Hebei, China

## Abstract

Myocardial fibrosis is the main morphological change of ventricular remodelling caused by cardiovascular diseases, mainly manifested due to the excessive production of collagen proteins. SRY-related high mobility group-box gene 9 (SOX9) is a new target regulating myocardial fibrosis. Bellidifolin (BEL), the active component of *G. acuta*, can prevent heart damage. However, it is unclear whether BEL can regulate SOX9 to alleviate myocardial fibrosis. The mice were subjected to isoproterenol (ISO) to establish myocardial fibrosis, and human myocardial fibroblasts (HCFs) were activated by TGF-*β*1 in the present study. The pathological changes of cardiac tissue were observed by HE staining. Masson staining was applied to reveal the collagen deposition in the heart. The measurement for expression of fibrosis-related proteins, SOX9, and TGF-*β*1 signalling molecules adopted Western blot and immunohistochemistry. The effects of BEL on HCFs, activity were detected by CCK-8. The result showed that BEL did not affect cell viability. And, the data indicated that BEL inhibited the elevations in *α*-SMA, Collagen I, and Collagen III by decreasing SOX9 expression. Additionally, SOX9 suppression by siRNA downregulated the TGF-*β*1 expression and prevented Smad3 phosphorylation, as supported by reducing the expression of *α*-SMA, Collagen I, and Collagen III. In vivo study verified that BEL ameliorated myocardial fibrosis by inhibiting SOX9. Therefore, BEL inhibited SOX9 to block TGF-*β*1 signalling activation to ameliorate myocardial fibrosis.

## 1. Introduction

Cardiovascular diseases (CVD) are ranked as the world's biggest killer because of their high morbidity and mortality and carry an increasing social and economic burden [1]. Myocardial fibrosis is the most common pathological change of CVD, which is the morphological feature of ventricular remodelling. This pathological cardiac remodelling presents the accumulation of excessive extracellular matrix proteins that are mainly type I and III collagen [2]. The increase in ventricular stiffness caused by collagen deposition is closely related to many harmful outcomes, such as heart failure and even cardiac death [2]. Therefore, inhibition of myocardial fibrosis would be a meaningful study for CVD treatment.

SRY-related high mobility group-box gene 9 (SOX9) is a highly conserved transcription factor family member. Early studies have illustrated the diverse functions of the SOX9 gene, including embryonic development [3], sex differentiation [4], and cartilage formation [5], and promoting tumor proliferation, invasion, metastasis, and progression [6–8]. Accumulating studies have confirmed that SOX9 could regulate collagen production, contributing to liver fibrosis, renal fibrosis, and cardiac fibrosis [9–12]. In cardiac fibroblasts, inhibition of SOX9 ameliorated cardiac fibrosis [12,13]. Numerous studies demonstrated that TGF-*β*1 could regulate SOX9 in the gastric cancer cell, kidney, and atrial fibroblasts [8,11,14], while another study showed that SOX9 could upregulate TGF-*β*1 expression in hepatic tissue of mice after IR injury [15]. However, the relationship between SOX9 and TGF-*β*1 signalling remains unclear in fibroblasts involved in myocardial fibrosis.

Bellidifolin (BEL) is a kind of xanthone compound from *Gentianella acuta* (Michx.) Hulten (*G. acuta*), which is used to treat heart diseases in the Inner Mongolia region. In the previous studies, BEL was shown to protect cardiomyocytes from oxidative damage and alleviate cardiac ischemia/reperfusion injury [16–18]. Our study demonstrates that BEL can relieve myocardial fibrosis by preventing TGF-*β*1/Smads signalling [19]. However, whether BEL-mediated inhibition of cardiac fibrosis is related to SOX9 remains to be explored. Here, we investigate the relationship between TGF-*β* signalling and SOX9 in cardiac fibroblast and explore the mechanism of BEL against myocardial fibrosis to provide a new strategy for heart failure therapy.

## 2. Materials and Methods

### 2.1. Drugs and Reagents

Isoproterenol (ISO) was supplied by Tokyo Chemical Industry Co., Ltd. (Japan). Drugs BEL and Trimetazidine (TMZ) used in the study were obtained from Chengdu Alfa Biotechnology (China) and Nanjing Zenkom Pharmaceutical Co., Ltd. (China), respectively. The staining kits for hematoxylin-eosin (HE) and Masson trichrome were provided by Servicebio Technology Co., Ltd. (China). Collagen I (cat. no.AF7001) and Collagen III (cat. no.AF0136) antibodies were offered by Affinity Biosciences (USA). Primary antibodies for *α*-SMA (cat. no. ab32575), SOX9 (cat. no. ab185966), Smad3 (cat. no. ab40854), phospho-Smad3 (S423 + S425) (cat. no. ab52903), and TGF-*β*1 (cat. no. ab92486) were supplied by Abcam Technology (United Kingdom). Glyceraldehyde 3-phosphate dehydrogenase (GAPDH, cat. no. 60004-1-Ig) was obtained from Wuhan Proteintech Biotechnology (China). HRP-conjugated antirabbit and antimouse IgG (cat. nos.ZdR-5306 and ZdR-5307) antibodies were offered by Beijing ZSGB Bioengineering Institute (China). DMEM medium was gained from Gibco (USA). The fetal bovine serum (FBS) and penicillin/streptomycin were provided by Biological Industries (Israel). Small interfering RNA (siRNA) was obtained from Shanghai GenePharma (China). Recombinant human TGF-*β*1 and Lipofectamine^TM^ 3000 reagents were supplied by Thermo Fisher Scientific (USA).

### 2.2. Culture Human Cardiac Fibroblasts (HCFs)

Human cardiac fibroblasts (HCFs) were provided by Shanghai Honsun Biological Technology Co., Ltd (China). HCFs were grown in a DMEM medium (Gibco, United States) with the addition of 10% FBS and 1% penicillin/streptomycin maintained at 37°C and in the condition of 5% CO_2_.

### 2.3. Cell Viability

Cell counting kit 8 (CCK-8) assay (MedChem Express, United States) was employed to evaluate cell viability following the manufacturer's instructions. In brief, HCFs were plated in 96-well plates with 5000 cells/well, grown for 24 h, and subsequently administrated with BEL (3.125, 6.25, 12.5, 25, 50, and 100 *μ*M) for 24 h. Then, the addition of CCK-8 solution (10 *μ*L/well) was applied to incubate HCFs at 37°C for 1 h. A microplate reader (Thermo Fisher Scientific, United States) detected the absorbance at 450 nm.

### 2.4. HCFs Grouping and Administration

DMSO was provided by Solarbio, China, and applied to dissolve BEL for a final concentration of 1/1,000. The HCFs were divided into five experimental groups: control plus DMSO, TGF-*β*1 plus DMSO, TGF-*β*1 plus BEL (12.5, 25, and 50 *µ*M) groups. When the cell density reached 60–70%, the HCFs were starved for 24 h and then intervened with TGF-*β*1 (10 ng/mL) or/and BEL for 24 h.

### 2.5. Transient Transfections

HCFs were transiently transfected with SOX9 siRNA using Lipofectamine TM 3000 reagent based on the manufacturer's instructions. HCFs were cultured in DMEM containing 10% FBS for 24 h after transfection and then starved for 12 h followed by treatment with or without TGF-*β*1 for 24 h.

### 2.6. Animals' Protocol

Kunming male mice (*n* = 60) were provided by the Laboratory Animal Center of Hebei Medical University (Shijiazhuang, China). Before the experiment, the mice were adaptively fed for 7 days before the experiment under a standardized environment, namely temperature (21 ± 1°C), humidity (55–60%), and a 12 h light and dark cycle obtaining food and water freely. Animal protocols were implemented on the basis of the relevant documents of the Animal Ethics Committee of the Hebei University of Chinese Medicine (no. DWLL201802).

According to the previously described method [19], the experimental mice were randomly divided into five groups, including the control group (Control), the model group (ISO), low-dose BEL intervention group (ISO + BEL 25 mg/kg), high-dose BEL intervention group (ISO + BEL 50 mg/kg), and trimetazidine (TMZ) intervention group (ISO + TMZ 20 mg/kg). Except for the control group, others were administered with 5 mg/kg ISO by subcutaneously injecting for 7 days to establish a model of myocardial fibrosis in mice. The day after ISO injection, the mice were given different BEL and TMZ by gavage for 21 days. The control and model groups were given the same volume of 0.05% sodium cellulose carboxylate solvent by gavage.

### 2.7. Histological Examination

Left ventricles were fixed using 4% paraformaldehyde for 24 h, embedded in paraffin, and cut into 4 *μ*m sections. The pathological changes of cardiac tissue were observed by HE staining, and the deposited collagen was examined using Masson trichrome staining based on the manufacturer's instructions. The results were visualised and photographed employing light microscopy (Leica DM4000B; Leica Microsystems, Wetzlar, Germany).

### 2.8. Western Blot Analysis

According to the previously described method [19], total protein extraction was applied with RIPA buffer, adding a protease inhibitor cocktail, phenylmethylsulfonyl fluoride, and phosphatase inhibitors. Protein concentration was measured using a bicinchoninic acid protein assay kit (Wuhan Servicebio Technology, Ltd., China). Equal amounts of protein were loaded into the wells of SDS-PAGE (10%) and separated. A semidry transfer printer (Bio-rad, United States) was used to transfer protein from gel to PVDF membranes. Nonfat milk (5%) was used to block nonspecific epitopes for 90 min. The membranes were incubated with primary antibodies for *α*-SMA, TGF-*β*1, Collagen I, Collagen III, SOX9, Smad3, phospho-Smad3, and GAPDH according to instructions overnight at 4°C. After washing three times with TBST, the membranes were incubated by the corresponding secondary antibodies labelled with HRP for 90 min at RT. The blot bands were visualised by the ECL (Invitrogen) reagents and Fusion FX5 Spectra (VilberLourmat, France). Each protein was quantified by transmittance densitometry using Image J 3.0 software (National Institute of Health).

### 2.9. Real-Time Fluorescent Quantitative PCR (qRT-PCR) Analysis

Total RNA from HCFs was extracted using Trizol reagent (Invitrogen). On the basis of the manufacturer's instructions, synthesised cDNAs, adopted the Reverse Transcription Kit, and were regarded as the template for qRT-PCR. CFX Connect Real-Time Detection System (Bio-Rad Laboratories, Inc., Singapore) was employed to quantify the results. GAPDH was used to normalise the target gene. The primers set for HCFs genes were as follows: GAPDH: forward, GGA TTT GGT CGT ATT GGG; reverse, GGA AGA TGG TGA TGG GAT T; SOX9: forward, CAC ACG CTG ACC ACG CTG AG; reverse, GCT GCT GCT GCT CGC TGT AG.

### 2.10. Statistical Analysis

SPSS 22.0 software was employed to conduct data analysis, and the measurement data were calculated using mean ± SEM. The differences in each group were compared using ANOVA, accompanied by Turkey's post hoc test. *p* < 0.05 is statistically considered as a significant difference.

## 3. Results

### 3.1. BEL-Downregulated TGF-*β*1 Induced the Increases of *α*-SMA, Collagen I, and Collagen III in HCFs

**Figure 1:**
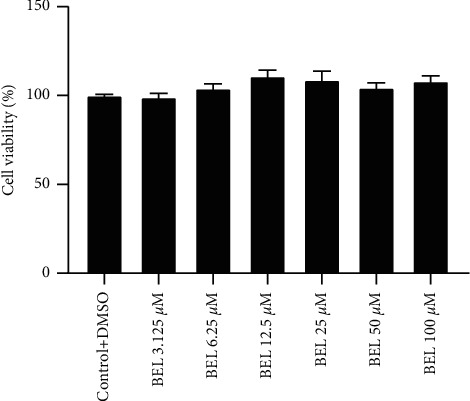
BEL did not affect HCF cells viability. HCFs were administered with different doses of BEL (3.125, 6.25, 12.5, 25, 50, and 100 *μ*M) for 24 h, and a CCK-8 assay was applied to investigate the cell viability.

**Figure 2:**
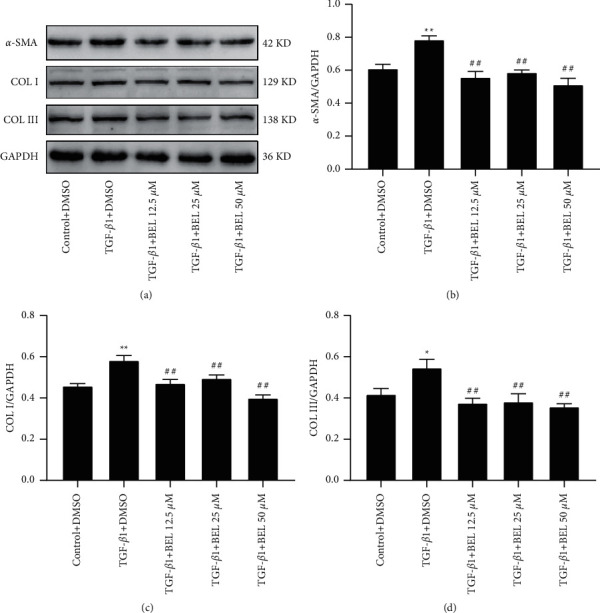
BEL prevented TGF-*β*1-increased the expression of *α*-SMA, Collagen I, and Collagen III. (a) Western blot examined the expression of *α*-SMA, Collagen I, and Collagen III. (b–d) The fold changes of the Western blot results for *α*-SMA, Collagen I, and Collagen III were analysed; GAPDH was used as a loading control (*n* = 3). Data were presented as the mean ± standard error of the mean (SEM). ^*∗*^*p* < 0.05 and ^*∗∗*^*p* < 0.01 vs. Control; ^##^*p* < 0.01 vs. TGF-*β*1.

We detected BEL's effect on cell viability in HCFs using the CCK-8 kit. The results illustrated that HCFs viability treated with different doses (3.125, 6.25, 12.5, 25, 50, and 100 *μ*M) of BEL for 24 h produced no statistical difference compared to the control group (Figure 1). Further, Western blot was employed to detect the change in *α*-SMA, Collagen I, and Collagen III by TGF-*β*1 administration and intervened in BEL for 24 h in HCFs. The results indicated that TGF-*β*1 significantly raised the expression of *α*-SMA, Collagen I, and Collagen III compared with the control group in HCFs. However, different doses of BEL intervention significantly downregulated the increases in the expression of *α*-SMA, Collagen I, and Collagen III (Figure 2). These results illustrated that BEL could downregulate TGF-*β*1 increased expression of *α*-SMA, Collagen I, and Collagen III in HCFs.

### 3.2. BEL Impeded the SOX9 Expression and Smad3 Phosphorylation Administered by TGF-*β*1 in HCFs

**Figure 3:**
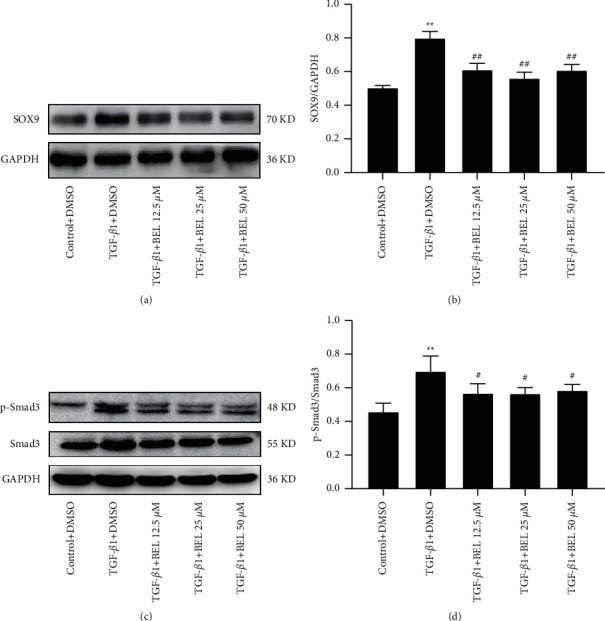
BEL reduced SOX9 expression and Smad3 phosphorylation. (a, c) TGF-*β*1-stimulated the expression of SOX9 and Smad3 and the level of Smad3 phosphorylation were analysed by Western blot. (b, d) Bar graphs presented fold changes of SOX9 and P-Smad3/Smad3. GAPDH was used as a loading control (*n* = 3). Data were presented as mean ± SEM. ^*∗∗*^*p* < 0.01 vs. Control; ^#^*p* < 0.05 and ^##^*p* < 0.01 vs. TGF-*β*1.

SOX9 is regarded as a new target regulating myocardial fibrosis. Western blot was applied to analyse SOX9 protein expression in TGF-*β*1-stimulated HCFs in the absence or presence of BEL. The results indicated that TGF-*β*1 elevated SOX9 expression in HCFs. However, intervention with BEL (12.5, 25, and 50 *μ*M) for 24 h prevented these elevations (Figures 3(a) and 3(b)).

Smad3 is the foremost TGF-*β*1 signalling molecule contributing to myocardial fibrosis. Western blot results verified that Smad3 phosphorylation was upregulated by TGF-*β*1 administration in HCFs, and downregulated by BEL intervention (Figures 3(c) and 3(d)). These data demonstrated that BEL could impede the SOX9 expression and prevent Smad3 phosphorylation in HCFs.

### 3.3. Knockdown of SOX9 Blocked TGF-*β*1/Smad3 Signalling in HCFs

**Figure 4:**
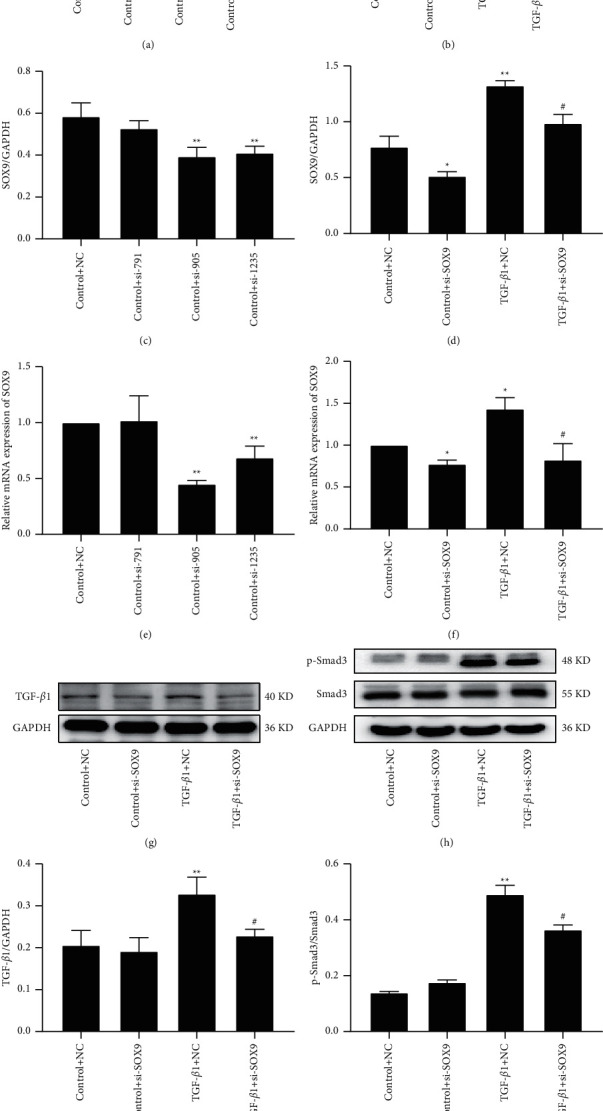
SOX9 knockdown suppressed TGF-*β*1/Samd3 pathway activation. (a, c, e) Western blot and qRT-PCR evaluated siRNA transfection efficiency, and bar graphs presented fold changes of SOX9 expression. GAPDH was used as a loading control (*n* = 3). (b, d, f) Western blot and qRT-PCR analysed the effect of SOX9-Homo-905 siRNA on SOX9 administrated by TGF-*β*1. (g, h) The effect of knockdown of SOX9 on the expression of TGF-*β*1 and Smad3 and the level of Smad3 phosphorylation in the presence of TGF-*β*1 were detected by western blot. (i, j) The fold changes of TGF-*β*1 and P-Smad3/Smad were presented by bar graphs. GAPDH was used as a loading control (*n* = 3). Data were presented as mean ± SEM. ^*∗*^*p* < 0.05 and ^*∗∗*^*p* < 0.01 vs. Control + NC; ^#^*p* < 0.05 vs. TGF-*β*1 + NC.

To ascertain the relationship between SOX9 and TGF-*β*1/Smad3 signalling, we adopted siRNA to block SOX9. The knockdown efficiency of SOX9 was evaluated by Western blot and qRT-PCR after transfection for 24 h with SOX9-Homo-791 siRNA, SOX9-Homo-905 siRNA, and SOX9-Homo-1235 siRNA. The results indicated that SOX9-Homo-905 siRNA had the highest knockdown efficiency of the three segments and was applied to study the relationship of SOX9 and TGF-*β*1/Smad3 in subsequent experiments (Figures 4(a), 4(c), and 4(e)).

Knockdown of SOX9 used by SOX9-Homo-905 siRNA decreased the expression of SOX9 induced by TGF-*β*1 (Figures 4(b), 4(d), and 4(f)). Also, Western blot showed that the exogenous TGF-*β*1-induced TGF-*β*1 elevation was inhibited by SOX9-Homo-905 siRNA (Figures 4(g) and 4(i)). Smad3 expression and phosphorylation were analysed by western blot. The results demonstrated that Smad3 phosphorylation was note worthily increased by TGF-*β*1, and SOX9-Homo-905 siRNA attenuated TGF-*β*1-increased Smad3 phosphorylation (Figures 4(h) and 4(j)). These results demonstrated that downregulation of SOX9 expression by SOX9 siRNA decreased TGF-*β*1/Smad3 signalling activity in HCFs.

### 3.4. Inhibition of SOX9 Reduced the Elevations in *α*-SMA, Collagen I, and Collagen III Administered by TGF-*β*1 in HCFs

**Figure 5:**
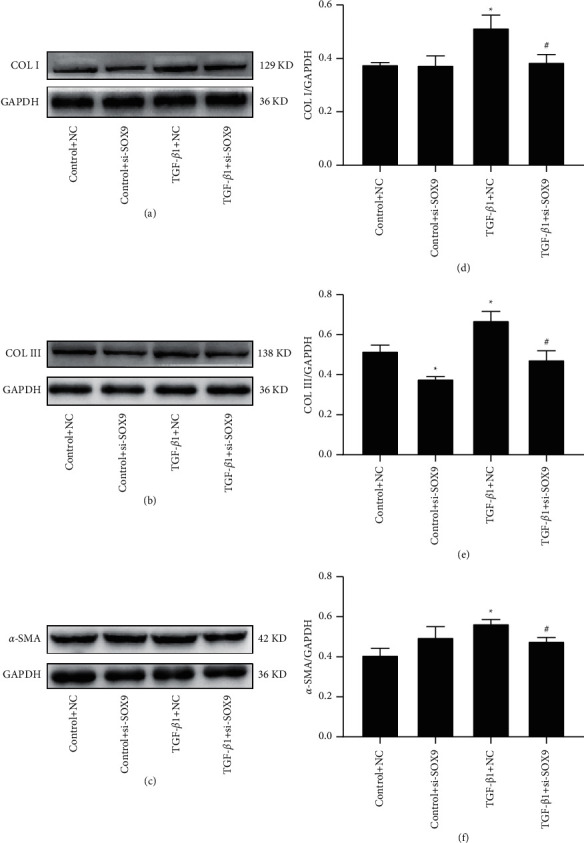
SOX9 knockdown inhibited TGF-*β*1-induced the expression of *α*-SMA, Collagen I, and Collagen III. (a–c) The expression of *α*-SMA, Collagen I, and Collagen III were assessed by Western blot. (d–f) Bar graphs presented fold changes of *α*-SMA, Collagen I, and Collagen III. GAPDH was used as a loading control (*n* = 3). Data were presented as the mean ± SEM. ^*∗*^*p* < 0.05 and ^*∗∗*^*p* < 0.01 vs. Control + NC; ^#^*p* < 0.05 vs. TGF-*β*1 + NC.

Fibrosis-related proteins, *α*-SMA, Collagen I, and Collagen III, were assessed by Western blot. The data demonstrated that the expression of *α*-SMA, Collagen I, and Collagen III were increased by exogenous TGF-*β*1 compared with the control group (Figure 5). While knockdown of SOX9 prevented the elevations in *α*-SMA, Collagen I, and Collagen III (Figure 5). These results illustrated that suppression of SOX9 could inhibit the elevations in *α*-SMA, Collagen I, and Collagen III stimulated by TGF-*β*1 in HCFs.

### 3.5. BEL Improved ISO-Induced Morphological Changes in Mice

**Figure 6:**
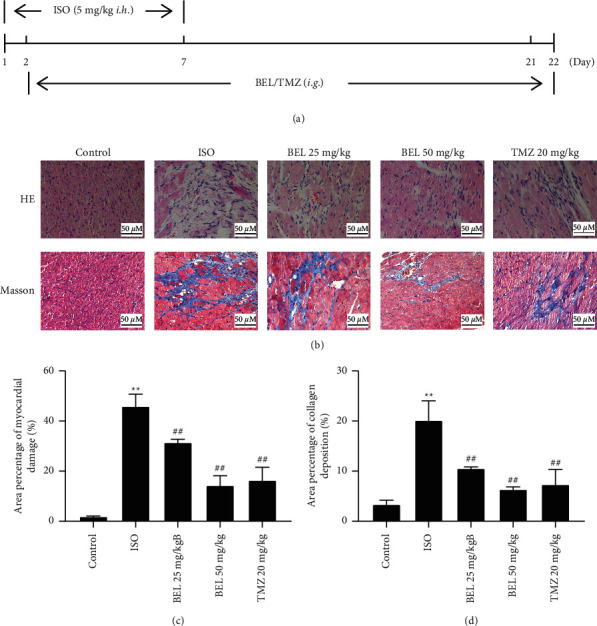
BEL ameliorated ISO-induced morphological changes in mice. (a) The flow diagram for the experimental design in mice. (b) HE and Masson staining examined morphological changes in mice. (c, d) Bar graphs showed the area of myocardial injury and the percentage of collagen deposition. ^*∗∗*^*p* < 0.01 vs. Control; ^##^*p* < 0.01 vs. ISO.

ISO-induced mice myocardial fibrosis model was established to observe the effect of BEL on myocardial fibrosis (Figure 6(a)). HE staining showed that the myocardial interstitial structure was complete, and the myocardial fibre morphology was regular in the control group (Figure 6(b)). Compared with the control group, the ISO group displayed myocardial fibre arrangement disorder, myocardial cell degeneration and necrosis, and much inflammatory cell infiltration. However, both the BEL low-dose and high-dose interventions significantly improved ISO-induced histopathological changes, and the TMZ intervention group also had the same results (Figures 6(b) and 6(c)).

Masson staining indicated that ISO resulted in a large amount of collagen deposition in the area of myocardial infarction. While treated with BEL and TMZ reduced ISO-increased collagen deposition (Figures 6(b) and 6(d)). These data verified that BEL could improve ISO-induced myocardial fibrosis in mice.

### 3.6. BEL Prevented the Elevations in Collagen I, Collagen III, and *α*-SMA by Blocking SOX9 in ISO-Induced Mice

**Figure 7:**
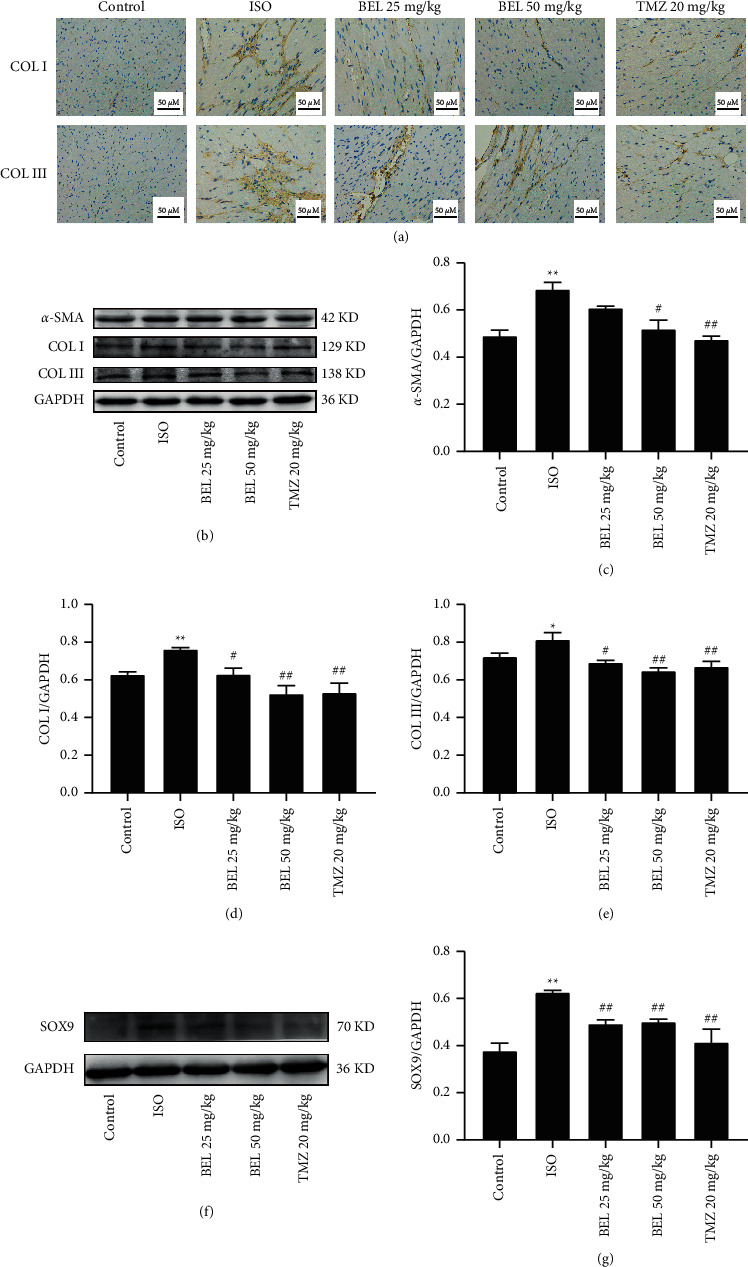
BEL prevented ISO-induced elevations in Collagen I, Collagen III, *α*-SMA, and SOX9 in mice. (a) Immunohistochemical staining examined the expression of Collagen I and Collagen III. (b) Western blot analysed the expression of *α*-SMA, Collagen I, and Collagen III. (c–e) The fold changes of *α*-SMA, Collagen I, and Collagen III were shown by bar graphs; GAPDH was used as a loading control (*n* = 3). (f) Detecting the expression of SOX9 using Western blot. (g) The fold changes of SOX9 were presented by bar graphs. GAPDH was used as a loading control (*n* = 3). Data were presented as mean ± SEM. ^*∗*^*p* < 0.05 and ^*∗∗*^*p* < 0.01 vs. Control; ^#^*p* < 0.05 and ^##^*p* < 0.01 vs. ISO.

To verify whether SOX9 is involved in myocardial fibrosis in vivo, the expression of SOX9, *α*-SMA, Collagen I, and Collagen III was evaluated. We found that ISO elevated the expression of *α*-SMA, Collagen I, and Collagen III compared to the control group, and SOX9 expression was also observably increased with ISO-induced collagen deposition (Figure 7). BEL treatment significantly prevented the elevations in *α*-SMA, Collagen I, and Collagen III. Moreover, SOX9 expression decreased with BEL treatment (Figure 7). These results revealed that BEL inhibited myocardial fibrosis through downregulating SOX9 expression in mice.

## 4. Discussion

In Mongolian medicine, *G. acuta* is one of the most common herbs in the Gentianaceae family used to treat hepatitis, jaundice, fever, and headache [20]. In Inner Mongolia, *G. acuta* has been applied to treat angina by the local herdsmen, and increasing studies have reported that it can protect the heart from injury [20]. We have verified that *G. acuta* and its xanthone compound BEL could alleviate myocardial fibrosis by preventing TGF-*β*1 signalling [19,21]. The present study further illuminated the mechanism of BEL on myocardial fibrosis in vivo and in vitro. The results demonstrated that BEL could alleviate cardiac structural disorder, reduce collagen deposition, and decrease the elevations in *α*-SMA, Collagen I, and Collagen III by downregulating SOX9 expression in vivo and in vitro. Additionally, blocking SOX9 reduced the TGF-*β*1 expression and prevented Smad3 phosphorylation. The research confirmed that BEL blocks SOX9 to regulate TGF-*β* signalling to ameliorate myocardial fibrosis.

SOX9 is a highly conserved member of the transcription factor family involved in the formation of cartilage and heart valves in mammals [22,23], contributing to the development of the pancreas, testes, liver, and other organs [24–28] and resulting in tumor proliferation, invasion, metastasis, and progression [6–8]. The report has confirmed that SOX9 is involved in collagen synthesis during heart development [23]. SOX9, an important regulator of ECM genes, plays a vital role in the pathological mechanisms of various diseases, such as liver fibrosis, glomerular sclerosis, and heart valve calcification [29–31]. Increasing attention has been paid to SOX9 in myocardial fibrosis. Recently, SOX9 was proposed as a potential therapeutic target for myocardial fibrosis because of ischemia-reperfusion injury [12]. Targeting SOX9 in fibroblasts could improve myocardial fibrosis by regulating AKT/GSK-3*β*/*β*-catenin [13]. In the present experiment, we confirmed that BEL could prevent the increase of SOX9 in vivo and in vitro. Further, we show that inhibiting SOX9 in HCFs decreased the elevations in *α*-SMA, Collagen I, and Collagen III.

TGF-*β*1/Smads in canonical signalling contribute to myocardial fibrosis. TGF-*β* is considered a key regulator of myocardial fibrosis. Present studies have demonstrated that the TGF-*β*1 signal is activated and involved in the process of myocardial injury repair and cardiac remodelling [32]. Smad3 is the most important signalling molecule in the canonical pathway [33]. The previous study also confirmed that BEL could prevent rat cardiac fibroblasts proliferation and activation by regulating TGF-*β*1/Smads, especially inhibiting TGF-*β*/Smad3 [19]. In this study, we again illustrate that BEL impedes TGF-*β*/Smad3 in HCFs to inhibit the expression of *α*-SMA, Collagen I, and Collagen III induced by TGF-*β*1.

Increasing evidence has demonstrated that there is a relation between SOX9 and TGF-*β*1. Single-Cell RNA Sequencing Analysis indicated that both TGF-*β* canonical signalling and SOX9 transcription factors could regulate the response of cardiac fibroblasts to cardiac injury [34]. Studies have shown that TGF-*β*1 can upregulate SOX9 expression to promote epithelial-to-mesenchymal transition (EMT) in cancer, atrial, and renal fibrosis [8,11,14]. Another study reported that the elevation of SOX9 could activate TGF-*β*1 to exacerbate hepatic ischemia/reperfusion (IR) injury [15]. This study also explores the relationship between SOX9 and TGF-*β*1 signalling in HCFs. The results confirmed that SOX9 expression was upregulated in HCFs treated with TGF-*β*1. And blocking SOX9 by siRNA could prevent the elevations in *α*-SMA, Collagen I, and Collagen III and alleviate the profibrotic role of TGF-*β*1. Knockdown of SOX9 could also downregulate the TGF-*β*1 expression and impede Smad3 phosphorylation. We also confirmed that BEL could downregulate SOX9 expression and prevent Smad3 phosphorylation administrated by TGF-*β*1 in HCFs. These results verified that TGF-*β*1 could regulate SOX9 expression and promote collagen deposition and fibrosis. Moreover, SOX9 can also regulate TGF-*β*1 expression and promote TGF-*β*1 signalling activation. Thus, our previous and present studies indicate that BEL plays an antimyocardial fibrosis role by inhibiting SOX9 and further blocking the TGF-*β*1 signalling pathway.

## 5. Conclusion

**Figure 8:**
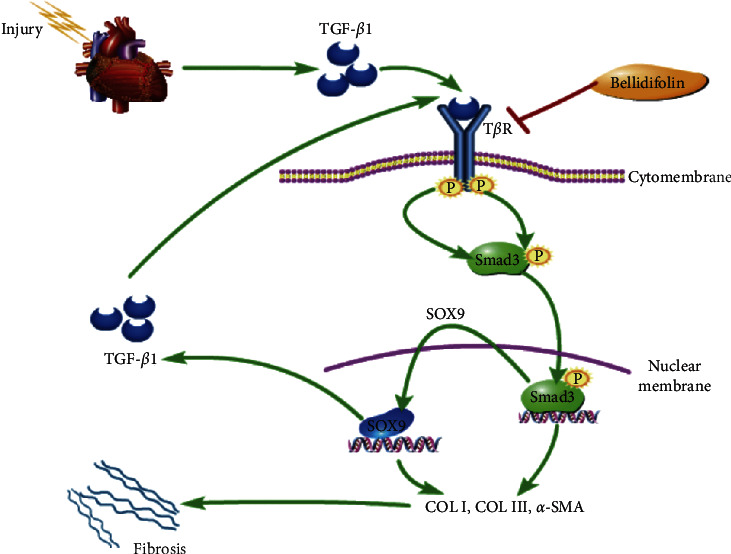
The proposed mechanism of BEL on myocardial fibrosis.

BEL appears to alleviate myocardial fibrosis by inhibiting SOX9 to suppress the elevations in *α*-SMA, Collagen I, and III. And inhibition of SOX9 downregulates the TGF-*β*1 expression and impedes Smad3 phosphorylation resulting in the downregulation of *α*-SMA, Collagen I, and Collagen III. In conclusion, BEL may provide a new therapeutic strategy by targeting SOX9 against myocardial fibrosis (Figure 8).

## Data Availability

The present data obtained from the findings are available from the corresponding author upon request.
